# Effects of probiotics on lipid metabolism, oxidation, and inflammation in coronary heart disease: a systematic review and meta-analysis

**DOI:** 10.3389/fnut.2025.1681230

**Published:** 2026-01-16

**Authors:** Xinyu Yang, Yunfeng Yu, Xiangning Huang, Juan Huang, Qin Xiang, Rong Yu

**Affiliations:** 1 School of Traditional Chinese Medicine, Hunan University of Chinese Medicine, Changsha, Hunan, China; 2 Department of Endocrinology, The First Hospital of Hunan University of Chinese Medicine, Changsha, Hunan, China

**Keywords:** coronary heart disease, inflammation, lipid metabolism, meta-analysis, oxidation, probiotics

## Abstract

**Objective:**

This meta-analysis and trial sequential analysis (TSA) aimed to evaluate the effects of probiotics on lipid metabolism, oxidative stress, and inflammation in patients with coronary heart disease (CHD).

**Methods:**

Six databases were systematically searched for relevant studies published before May 1, 2025. Basic study characteristics, outcome data, and risk of bias were extracted. Meta-analyses were performed using RevMan 5.3, TSA was conducted using TSA 0.9.5.10 beta, and publication bias was assessed using Egger’s test.

**Results:**

Eight randomized controlled trials involving 296 patients were included. Compared with the placebo, probiotics significantly reduced the low-density lipoprotein cholesterol (LDL-C) [mean difference (MD) −11.07 mg/dL, 95% confidence interval (CI) −19.94 to −2.20], malondialdehyde [standardized mean difference (SMD) −0.57, 95% CI −1.01 to −0.13], high-sensitivity C-reactive protein (MD −0.81 ng/mL, 95% CI −1.31 to −0.30), Toll-like receptor 4 (MD −4.13 ng/mL, 95% CI −5.39 to −2.88), and interleukin-6 (MD −3.22 ng/mL, 95% CI −4.01 to −2.44). Meanwhile, they increased the high-density lipoprotein cholesterol (HDL-C) (MD 2.79 mg/dL, 95% CI 0.95 to 4.63), glutathione (MD 104.66 μmol/L, 95% CI 53.74 to 155.58), and total antioxidant capacity (MD 69.51 mmol/L, 95% CI 44.64 to 94.38). However, the effects on LDL-C and HDL-C were not robust in sensitivity analyses, and neither outcome reached the minimal clinically important difference, indicating that lipid modulation is not clinically meaningful. Additionally, no significant differences were observed in very low-density lipoprotein cholesterol, total cholesterol, triglycerides, tumor necrosis factor-alpha and adverse event rate. TSA confirmed the robustness of all significant outcomes except for malondialdehyde, and Egger’s tests revealed no significant publication bias.

**Conclusion:**

Probiotics exert moderate anti-inflammatory and antioxidative effects, supporting their potential as an complementary strategy for CHD. In contrast, their influence on lipid metabolism appears uncertain and clinically negligible. Given the limited certainty of evidence and the geographic concentration of available trials, high-quality multicenter studies are required to confirm these findings.

## 1 Introduction

Coronary heart disease (CHD) is a group of cardiovascular disorders caused by coronary atherosclerosis, leading to arterial narrowing, spasm, or obstruction and ultimately resulting in myocardial ischemia (1, 2). According to the Global Burden of Disease Study, cardiovascular diseases are the leading causes of death and disability worldwide (3). Among them, ischemic heart disease accounts for 13% of all global deaths (4), with CHD being one of its predominant forms. The pathogenesis of CHD is multifactorial, primarily involving dyslipidemia, inflammation, and oxidative stress (5, 6). Dysregulated lipid metabolism lays the foundation for CHD, while inflammation and oxidative stress promote vascular endothelial injury and facilitate low-density lipoprotein cholesterol (LDL-C) deposition within the arterial wall (7, 8). Together, these interconnected mechanisms drive disease progression. Current treatment strategies for CHD include lifestyle modifications, revascularization procedures, and pharmacological interventions, such as lipid-lowering agents, antiplatelet drugs, and β-blockers (9, 10). Although these treatments improve clinical outcomes, they cannot reverse atherosclerosis and may cause adverse effects (11). For instance, statins can cause myalgia and, in rare cases, rhabdomyolysis, and may increase the risk of liver injury (12). Additionally, aspirin may lead to mucosal erosion and ulcers, thereby raising the risk of gastrointestinal bleeding (13). Therefore, exploring safe and effective complementary therapies is urgently needed to improve the prognosis and quality of life in CHD patients.

Emerging evidence highlights a critical role of gut microbiota dysbiosis in the pathogenesis of cardiovascular diseases (14). The intestinal epithelium serves as a selective barrier that maintains host–microbiota homeostasis. Disruption of epithelial integrity increases intestinal permeability, allowing bacterial components such as lipopolysaccharide to translocate into the circulation and trigger, systemic inflammation and endothelial dysfunction (15). Gut dysbiosis also alters microbial metabolic activity, resulting in reduced production of anti-inflammatory metabolites such as short-chain fatty acids and greater generation of pro-atherogenic compounds such as trimethylamine N-oxide (16). These changes enhance oxidative stress, activate inflammatory pathways including the NOD-like receptor family pyrin domain-containing 3 (NLRP3) inflammasome, and impair vascular homeostasis (16). Therefore, restoring microbial balance through microbiota-targeted interventions such as probiotics, prebiotics, or dietary modulation has gained increasing attention (17, 18). A recent meta-analysis reported that probiotic supplementation significantly improved glycemic control and lipid profiles in patients with CHD, providing preliminary evidence of potential clinical benefit (17). However, that analysis included participants who received probiotics in combination with selenium or vitamin D, both of which have intrinsic anti-inflammatory and antioxidant properties. These confounding factors may have compromised the robustness and reliability of the findings. In addition, inflammation- and oxidative stress-related outcomes were not comprehensively evaluated. Hence, a more rigorous and systematic evaluation is warranted to clarify the independent effects of probiotics on lipid metabolism, inflammation, and oxidative stress in patients with CHD.

Meta-analysis synthesizes evidence from multiple independent studies to estimate an overall effect size, whereas trial sequential analysis (TSA) helps control random errors and assess the robustness of cumulative findings (19, 20). This study aims to assess the effects of probiotics on lipid metabolism, oxidative stress, and inflammation in patients with CHD through meta-analysis and TSA, while also reporting on their safety. This approach addresses limitations of prior studies and provides high-quality evidence for the clinical application of probiotics in CHD. The graphical abstract is shown in Supplementary Figure S1.

## 2 Methods

This meta-analysis was performed following the guidelines established by the Preferred Reporting Items for Systematic Reviews and Meta-Analyses (PRISMA) (21). The primary objective was to assess the impact of probiotics on lipid metabolism, oxidative stress, and inflammation in patients with CHD.

### 2.1 Inclusion and exclusion criteria

#### 2.1.1 Inclusion criteria

(i) Participants: Individuals diagnosed with CHD, regardless of sex, age, ethnicity, or comorbidity status. Comorbidities were allowed because they commonly coexist with CHD in real-world clinical practice and reflect the typical CHD patient population. (ii) Interventions: The experimental group received probiotics or synbiotics in addition to standard treatment for CHD. Given that synbiotics are widely used in clinical practice and share similar mechanisms of action with probiotics, they were included as part of the intervention. (iii) Comparisons: The control group received the placebo alongside standard medical treatment for CHD. (iv) Outcomes: Blood lipid outcomes included LDL-C, very low-density lipoprotein cholesterol (VLDL-C), high-density lipoprotein cholesterol (HDL-C), total cholesterol (TC), and triglycerides (TG). Oxidation outcomes included glutathione (GSH), malondialdehyde (MDA), and total antioxidant capacity (TAC). Inflammatory outcomes included hs-CRP, Toll-like receptor 4 (TLR4), tumor necrosis factor-alpha (TNF-α), and interleukin-6 (IL-6). Among them, LDL-C was set as the primary efficacy outcome. The safety outcome was the adverse event rate (AER). (v) Study design: Only randomized controlled trials (RCTs) were included in this analysis.

#### 2.1.2 Exclusion criteria

(i) Duplicate publications: Studies that presented the same data in multiple articles were excluded to prevent redundancy. (ii) Abstract-only publications: Studies available solely as abstracts or conference proceedings without full-text access were excluded. (iii) Incomplete or ambiguous data: Studies that did not provide sufficient data or presented unclear outcomes were excluded. (iv) Inappropriate statistical methods: Studies that utilized incorrect statistical techniques, which could not be rectified, were excluded from the analysis.

### 2.2 Literature search

The comprehensive search was conducted across six databases: PubMed, Embase, Cochrane Library, Web of Science, MEDLINE, and ClinicalTrials.gov. The search strategy was focused on Title/Abstract or Topic, utilizing the following terms: ((Coronary Disease OR Coronary Diseases OR Coronary Heart Disease OR Coronary Heart Diseases OR Coronary Artery Disease OR Coronary Artery Diseases OR Myocardial Infarction OR Heart Attack OR Heart Attacks OR Myocardial Infarct OR Myocardial Infarcts) AND (Probiotic OR Probiotics OR *Bifidobacterium* OR *Bifidobacteria* OR *Bacillus bifida* OR Yeast OR *Saccharomyces cerevisiae* OR *Saccharomyces italicus* OR *Saccharomyces oviformis* OR *S. cerevisiae* OR *S. cerevisiae* OR *Saccharomyces uvarum* var. *melibiosus* OR *Candida robusta* OR *Saccharomyces capensis* OR *Lactobacillus acidophilus* OR *Lactobacillus amylovorus* OR Lactobacill OR lactic acid bacteria OR *Clostridium butyricum* OR Bacillus OR Natto Bacteria OR *Streptococcus thermophiles* OR Enterococcus)). The search covered the period from the inception of the databases until May 1, 2025, with no restrictions on language, country, or other factors. Additionally, reference lists from relevant reviews and included articles were manually screened to enhance the comprehensiveness of the search.

### 2.3 Study selection process

All identified records were imported into Endnote X9 software, where duplicates were removed automatically and verified manually. Subsequently, XY and YY independently evaluated the titles and abstracts based on the established inclusion and exclusion criteria. Then, full-text articles of potentially eligible studies were retrieved for independent assessment by both reviewers. Any disagreements were resolved through discussion or by XH. The PRISMA flow diagram was utilized to illustrate the study selection process.

### 2.4 Data extraction

The standardized form for data extraction was created. The information collected included: authors, publication year, country, sample size, age, sex, weight, intervention, comparison, outcomes measured, funding sources, and potential conflicts of interest. Data extraction was performed independently by XY and YY, and any discrepancies were discussed and resolved. Authors were contacted for clarification or missing data when necessary.

### 2.5 Risk of bias assessment

The methodological quality of the included studies was assessed independently by XY and YY using the Cochrane Risk of Bias (RoB 1.0) tool. The evaluation focused on several domains, including sequence generation, allocation concealment, blinding of participants and personnel, blinding of outcome assessment, incomplete outcome data, selective reporting, and other biases. Each domain was classified as low, high, or unclear risk. An overall judgment regarding the risk of bias was provided, with any disagreements resolved through discussion or by XH.

### 2.6 Data synthesis and statistical analysis

The meta-analysis was performed using RevMan 5.3 software. For dichotomous outcomes, such as AER, the risk ratio (RR) with 95% confidence interval (CI) was calculated. For continuous outcomes, including LDL-C, VLDL-C, HDL-C, TC, TG, GSH, MDA, TAC, hs-CRP, TLR4, TNF-α, and IL-6, the mean difference (MD) or standardized mean difference (SMD) with 95% CI was utilized, depending on the measurement tools employed. According to Cohen’s rule of thumb (22), an SMD of 0.2, 0.5, and 0.8 represents a small, moderate, and large effect size, respectively. Heterogeneity among studies was assessed using the Cochran’s *Q* test and *I*^2^ statistic. For the Cochran’s *Q* test, a *p* ≥ 0.10 indicated that the observed differences among study results were likely due to chance, suggesting minor heterogeneity, whereas *p* < 0.10 suggested significant heterogeneity. An *I*^2^ < 50% indicated minor heterogeneity, while *I*^2^ ≥ 50% suggested high heterogeneity. When the *p* ≥ 0.10 and *I*^2^ < 50%, a fixed-effects model was applied; otherwise, a random-effects model was applied.

Subsequently, the sensitivity analysis was conducted to evaluate the robustness of the meta-analysis results. The impact of studies with high risk of bias, small sample sizes, or extreme effect sizes was assessed by excluding each study individually. Consistent results across various sensitivity analyses indicated the reliability of the overall findings. Additionally, the pre-specified subgroup analysis based on clinical factors such as the source of participants, female ratio, average age, weight, probiotic dosage, and treatment duration was performed to assess the influence of clinical heterogeneity on the primary outcome.

Furthermore, the TSA was conducted using TSA 0.9.5.10 beta software to account for random errors and evaluate the reliability of cumulative evidence. The information size was determined based on an anticipated effect size, *α* = 5%, and power of 80%. TSA monitoring boundaries were applied to ascertain whether the cumulative evidence was sufficient for firm conclusions or whether additional trials were needed. Results were considered conclusive when the *Z*-curve crossed the monitoring boundary.

### 2.7 Publication bias assessment

Publication bias was evaluated quantitatively using Egger’s test, which evaluates funnel plot asymmetry by regressing the standard normal deviate of the effect size on its precision. A *p* < 0.05 was considered indicative of potential publication bias. This approach allows for a more objective detection of bias in the absence of funnel plot visualization.

### 2.8 Certainty of evidence

The certainty of evidence for each outcome was evaluated using the Grading of Recommendations, Assessment, Development, and Evaluation (GRADE) system, considering risk of bias, inconsistency, indirectness, imprecision, and publication bias. Each outcome was classified as high, moderate, low, or very low quality, which informed the confidence in the pooled estimates and the recommendations derived from them.

## 3 Results

### 3.1 Study selection

**Figure 1:**
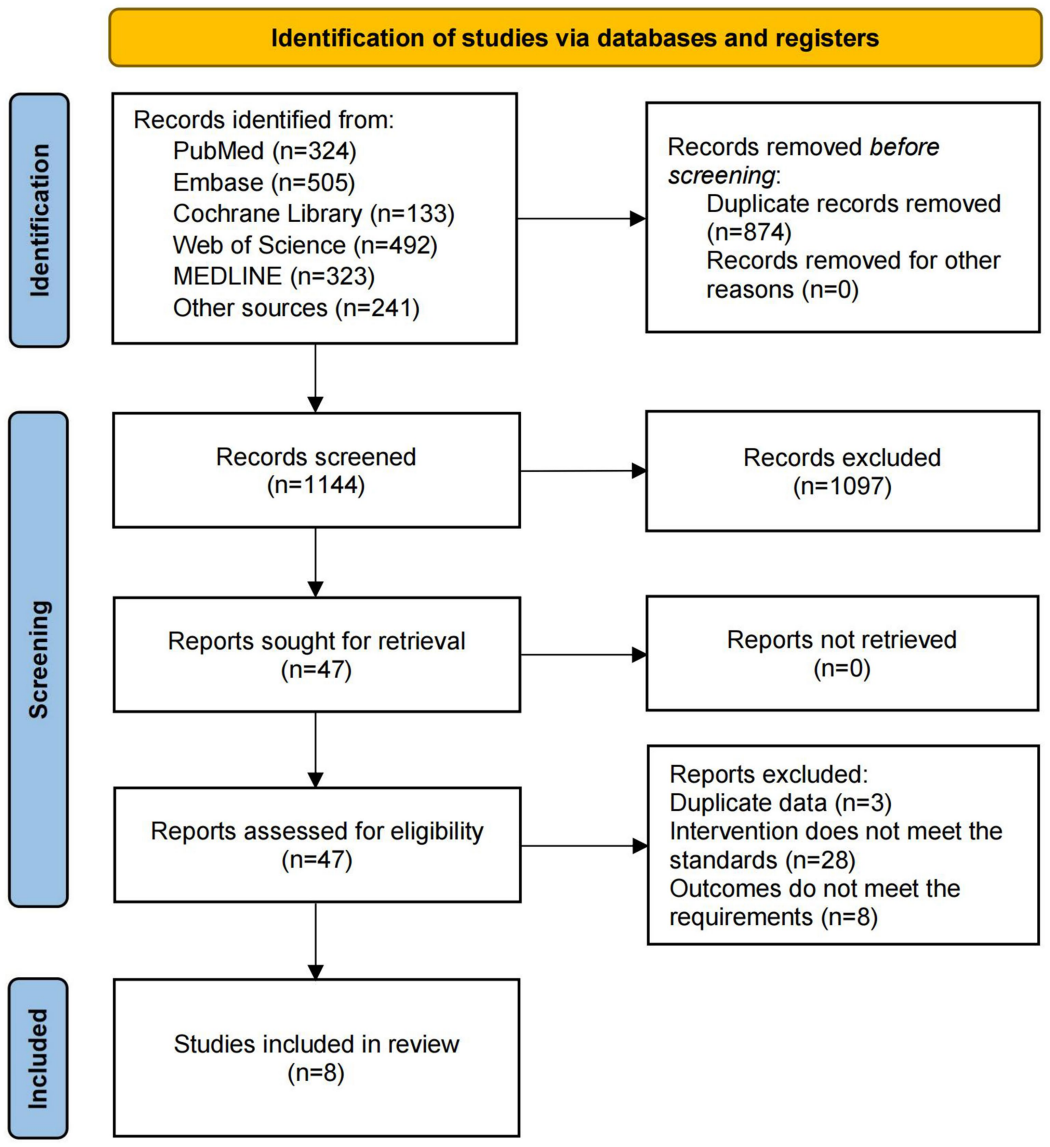
Literature screening process.

The comprehensive search across multiple databases identified a total of 2,018 records: 324 from PubMed, 505 from Embase, 133 from the Cochrane Library, 492 from Web of Science, 323 from MEDLINE, 221 from ClinicalTrials.gov, and 20 from reference lists. After removing 874 duplicates, the remaining 1,144 records were screened based on titles and abstracts, leading to the exclusion of 1,097 studies due to irrelevance. Full texts of the remaining articles were then assessed for eligibility, resulting in the exclusion of 39 studies: 3 due to duplicated data, 28 for not meeting intervention criteria, and 8 for not satisfying the outcome measures. Ultimately, 8 studies (23–30) were included in this meta-analysis. The detailed flow diagram is presented in Figure 1.

### 3.2 Basic characteristics

This meta-analysis included eight published articles (23–30), as shown in Table 1. These studies were published between 2017 and 2024, with half of them appearing within the past 5 years. One study [Sun et al. (29)] was conducted in China, while the others [Farrokhian et al. (23), Liu et al. (24), Moludi et al. (25), Moludi et al. (26), Moludi et al. (27), Raygan et al. (28), and Tajabadi-Ebrahimi et al. (30)] were carried out in Iran. To prevent inflated sample size and precision caused by overlapping populations, participant recruitment periods and registry identifiers were carefully examined. Two article pairs originated from the same trials and therefore involved identical participants: Farrokhian et al. (23) and Tajabadi-Ebrahimi et al. (30) both reported findings from IRCT201503025623N37 (*n* = 60), while Moludi et al. (25) and Moludi et al. (26) were based on IRCT20121028011288N15 (*n* = 44). Each pair was treated as a single trial, and the corresponding participant cohorts were counted only once. Additionally, Liu et al. (24) and Moludi et al. (27) both stemmed from IRCT20180712040438N4; although their experimental groups differed, they shared the same control participants. Following Cochrane methodological recommendations, the shared control group was evenly split to avoid double-counting. The remaining trials, including Raygan et al. (28) (IRCT2017082733941N5, *n* = 60) and Sun et al. (29) (ChiCTR1800017162, *n* = 60), were unique with no involve population overlap. After accounting for these issues, five distinct RCTs involving 296 participants were included in the quantitative synthesis.

**Table 1:** Basic characteristics of included studies.

Study	Diagnosis	Source	Sample (E/C)	Female (%)	Age (years)	Intervention	Comparison	Treatment duration (weeks)
Farrokhian et al. (23)	CHD with T2DM and BMI ≥25 kg/m^2^	Iran	30/30	63.3	64.1	*L. acidophilus*, *L. casei*, and *B. bifidum* 2 × 10^9^ CFU/day and inulin 800 mg qd	Placebo	12
Liu et al. (24)	CHD	Iran	24/24	35.4	50.5	*L. rhamnosus* 1.9 × 10^9^ CFU/day and inulin 15 mg qd	Placebo	8
Moludi et al. (25)	MI after successful PCI with BMI ≥25 kg/m^2^	Iran	22/22	6.8	52.6	*L. rhamnosus* 1.6 × 10^9^ CFU/day	Placebo	12
Moludi et al. (26)	MI after successful PCI with BMI ≥25 kg/m^2^	Iran	22/22	6.8	56.9	*L. rhamnosus* 1.6 × 10^9^ CFU/day	Placebo	12
Moludi et al. (27)	CHD	Iran	24/24	35.4	51.5	*L. rhamnosus* 1.9 × 10^9^ CFU/day	Placebo	8
Raygan et al. (28)	CHD with T2DM	Iran	30/30	/	61.3	*B. bifidum*, *L. casei*, and *L. acidophilus* 2 × 10^9^ CFU/day	Placebo	12
Sun et al. (29)	CHD	China	36/24	43.3	65.6	*B. lactis* 3 × 10^10^ CFU/day	Placebo	24
Tajabadi-Ebrahimi et al. (30)	CHD with T2DM and BMI ≥25 kg/m^2^	Iran	30/30	/	64.1	*L. acidophilus*, *L. casei*, and *B. bifidum* 2 × 10^9^ CFU/day and inulin 800 mg qd	Placebo	8

E, experimental group; C, control group; CHD, coronary heart disease; T2DM, type 2 diabetes mellitus; BMI, body mass index. There were no significant differences in female ratio, average age, or disease duration between the experimental and control groups in each included study.

The proportion of female participants ranged from 6.8 to 63.6%. Specifically, the female ratio was 6.8% in Moludi et al. (25) and Moludi et al. (26), 35.4% in Liu et al. (24) and Moludi et al. (27), 43.3% in Sun et al. (29), and 63.3% in Farrokhian et al. (23). The mean age of participants ranged from 50.5 to 65.6 years. Liu et al. (24), Moludi et al. (25), Moludi et al. (26), and Moludi et al. (27) enrolled participants with a mean age between 50 and 60 years, whereas Farrokhian et al. (23), Raygan et al. (28), and Tajabadi-Ebrahimi et al. (30) included participants aged between 60 and 70 years.

Regarding probiotic formulations, five studies [Liu et al. (24), Moludi et al. (25), Moludi et al. (26), Moludi et al. (27), and Sun et al. (29)] administered single-strain probiotics, while three studies [Farrokhian et al. (23), Raygan et al. (28), and Tajabadi-Ebrahimi et al. (30)] employed multi-strain combinations. Specifically, Liu et al. (24), Moludi et al. (25), Moludi et al. (26), and Moludi et al. (27) used *Lactobacillus rhamnosus*; Sun et al. (29) used *Bifidobacterium lactis*; Farrokhian et al. (23) administered a combination of *Lactobacillus acidophilus*, *Lactobacillus casei*, and *Bifidobacterium bifidum*; Raygan et al. (28) used *Bifidobacterium bifidum*, *Lactobacillus casei*, and *Lactobacillus acidophilus*; and Tajabadi-Ebrahimi et al. (30) used *Lactobacillus acidophilus*, *Lactobacillus casei*, and *Bifidobacterium bifidum*.

The treatment duration varied from 8 to 24 weeks. Liu et al. (24), Moludi et al. (27), and Tajabadi-Ebrahimi et al. (30) administered probiotics for 8 weeks; Farrokhian et al. (23), Moludi et al. (25), Moludi et al. (26), and Raygan et al. (28) for 12 weeks; and Sun et al. (29) for 24 weeks. Further detailed characteristics of the included studies are summarized in Table 1.

### 3.3 Risk of bias

**Figure 2:**
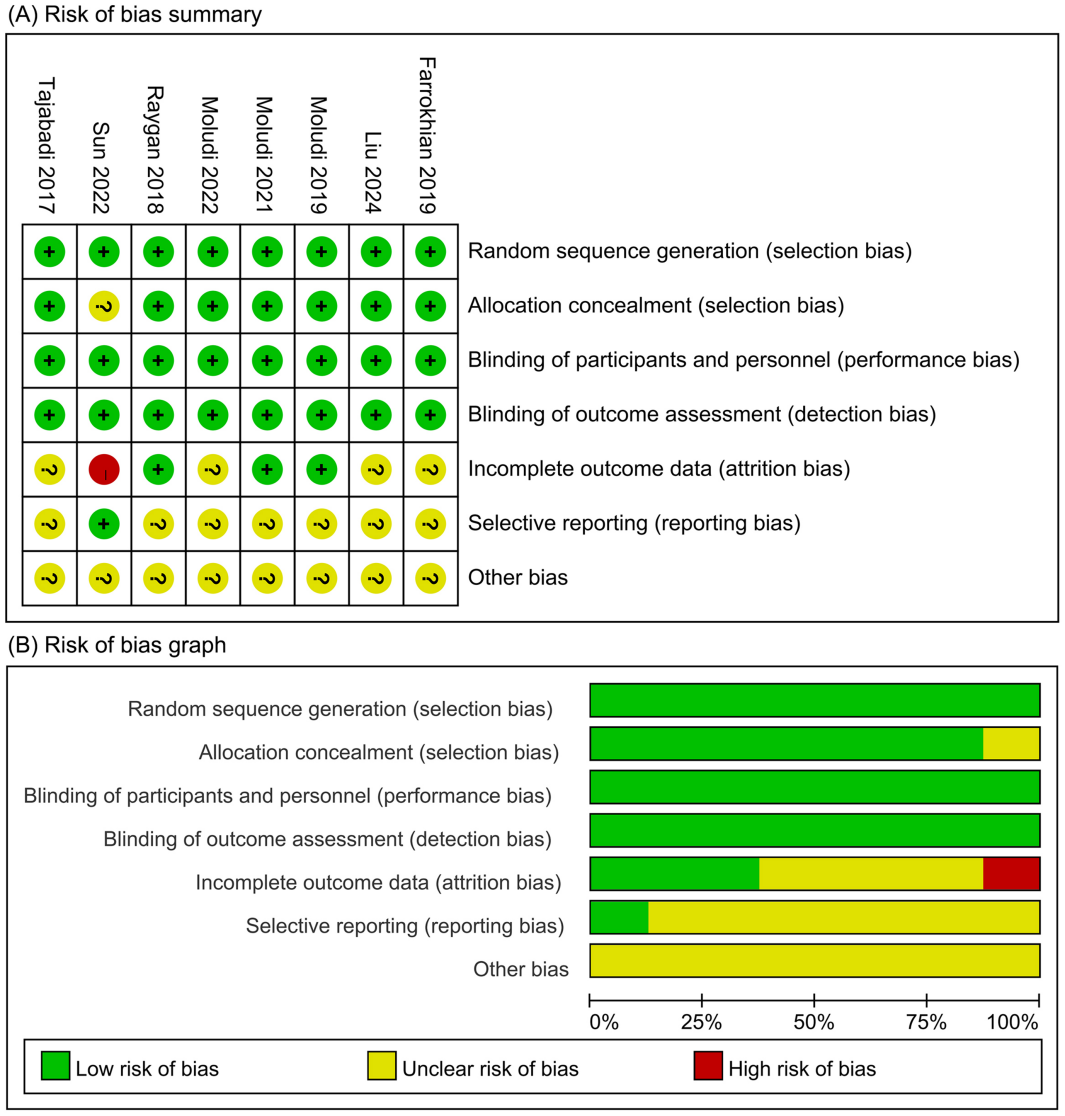
Risk assessment of bias: **(A)** Risk of bias summary; **(B)** Risk of bias graph.

The risk-of-bias assessment was conducted following the RoB 1.0 tool, as shown in Figure 2. For random sequence generation, all included studies reported using computer-generated randomization sequences, indicating a low risk of bias. Regarding allocation concealment, Sun et al. (29) described sequence generation but did not clarify how allocation was concealed, resulting in an unclear risk judgment for this domain, whereas the other trials provided detailed information and were judged as low risk. For blinding of participants and personnel, all studies employed a placebo-controlled design, suggesting a low risk of performance bias. Blinding of outcome assessment was considered low risk across studies because all outcomes were objective laboratory measures, such as lipid profiles and inflammatory and oxidative markers. In terms of incomplete outcome data, Sun et al. (29) reported dropout rates >20%, leading to a high risk of attrition bias, while for Farrokhian et al. (23), Liu et al. (24), Moludi et al. (27), and Tajabadi-Ebrahimi et al. (30), dropouts were <20%, but the potential impact on outcomes was not reported, resulting in an unclear risk judgment. Regarding selective reporting, although all trials provided clinical trial registration numbers, the registry entries for Farrokhian et al. (23), Liu et al. (24), Moludi et al. (25), Moludi et al. (26), Moludi et al. (27), Raygan et al. (28), and Tajabadi-Ebrahimi et al. (30) were not publicly accessible, preventing verification of prespecified outcomes and resulting in an unclear risk judgment. In contrast, Sun et al. (29) had a publicly accessible registry with complete prespecified outcomes, indicating low risk. Finally, for other sources of bias, insufficient evidence was available to exclude additional potential risks, and this domain was therefore judged as unclear.

### 3.4 Meta-analysis

#### 3.4.1 Blood lipid outcomes

##### 3.4.1.1 LDL-C

**Figure 3:**
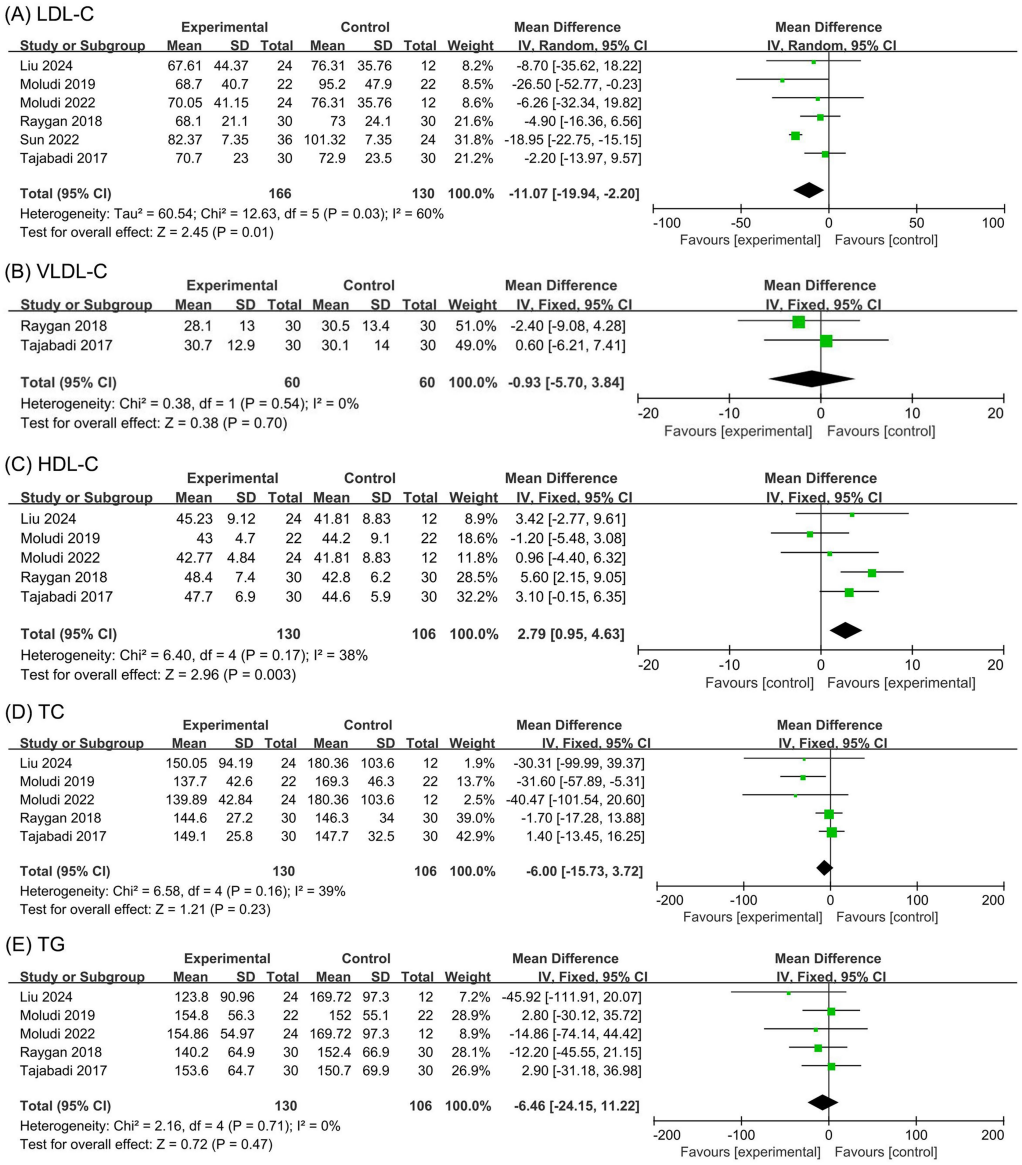
Forest plots of the meta-analysis on blood lipid outcomes: **(A)** LDL-C; **(B)** VLDL-C; **(C)** HDL-C; **(D)** TC; **(E)** TG. LDL-C, low-density lipoprotein cholesterol; VLDL-C, very low-density lipoprotein cholesterol; HDL-C, high-density lipoprotein cholesterol; TC, total cholesterol; TG, triglycerides.

The meta-analysis for LDL-C levels included six studies (24, 25, 27–30) with 296 participants. The Cochran’s *Q* test and *I*^2^ statistic indicated high heterogeneity (*p* = 0.03, *I*^2^ = 60%). The results demonstrated that the probiotic group significantly reduced LDL-C levels compared to the placebo group (MD −11.07 mg/dL, 95% CI −19.94 to −2.20, *p* = 0.01), as shown in Figure 3A. However, this reduction did not reach the minimal clinically important difference (MCID) of 38.67 mg/dL for LDL-C, suggesting that the observed change lacks clinical relevance. More importantly, LDL-C levels exhibited considerable heterogeneity and the effect was found to be unstable in the sensitivity analysis, as the significant reduction disappeared after excluding the study by Sun et al. (29). This instability compromises the robustness of the findings and lowers the certainty of evidence.

##### 3.4.1.2 VLDL-C

The meta-analysis for VLDL-C levels included two studies (28, 30) with 120 participants. The Cochran’s *Q* test and *I*^2^ statistic indicated minor heterogeneity (*p* = 0.54, *I*^2^ = 0). The results indicated no statistically significant difference in VLDL-C levels between the probiotic and placebo groups (MD −0.93 mg/dL, 95% CI −5.70 to 3.84, *p* = 0.70), as illustrated in Figure 3B.

##### 3.4.1.3 HDL-C

The meta-analysis for HDL-C levels comprised five studies (24, 25, 27, 28, 30) with 236 participants. The Cochran’s *Q* test and *I*^2^ statistic indicated minor heterogeneity (*p* = 0.17, *I*^2^ = 38%). The results demonstrated a significant increase in HDL-C levels in the probiotic group compared to the placebo group (MD 2.79 mg/dL, 95% CI 0.95 to 4.63, *p* = 0.003), as depicted in Figure 3C. However, this improvement did not meet the MCID threshold of 19.34 mg/dL for HDL-C, indicating limited clinical significance. Furthermore, sensitivity analysis revealed that the HDL-C result was unstable, as the significance was lost after excluding the study by Raygan et al. (28). This finding weakens the reliability of the pooled result and further reduces the certainty of evidence in the GRADE evaluation.

##### 3.4.1.4 TC

The meta-analysis for TC levels included five studies (24, 25, 27, 28, 30) with 236 participants. The Cochran’s *Q* test and *I*^2^ statistic indicated minor heterogeneity (*p* = 0.16, *I*^2^ = 39%). The results showed no significant difference in TC levels between the probiotic and placebo groups (MD −6.00 mg/dL, 95% CI −15.73 to 3.72, *p* = 0.23), as shown in Figure 3D.

##### 3.4.1.5 TG

The meta-analysis for TG levels included five studies (24, 25, 27, 28, 30) with 236 participants. The Cochran’s *Q* test and *I*^2^ statistic indicated minor heterogeneity (*p* = 0.71, *I*^2^ = 0). The results revealed no statistically significant difference in TG levels between the probiotic and placebo groups (MD −6.46 mg/dL, 95% CI −24.15 to 11.22, *p* = 0.47), as illustrated in Figure 3E.

#### 3.4.2 Oxidation outcomes

##### 3.4.2.1 GSH

**Figure 4:**
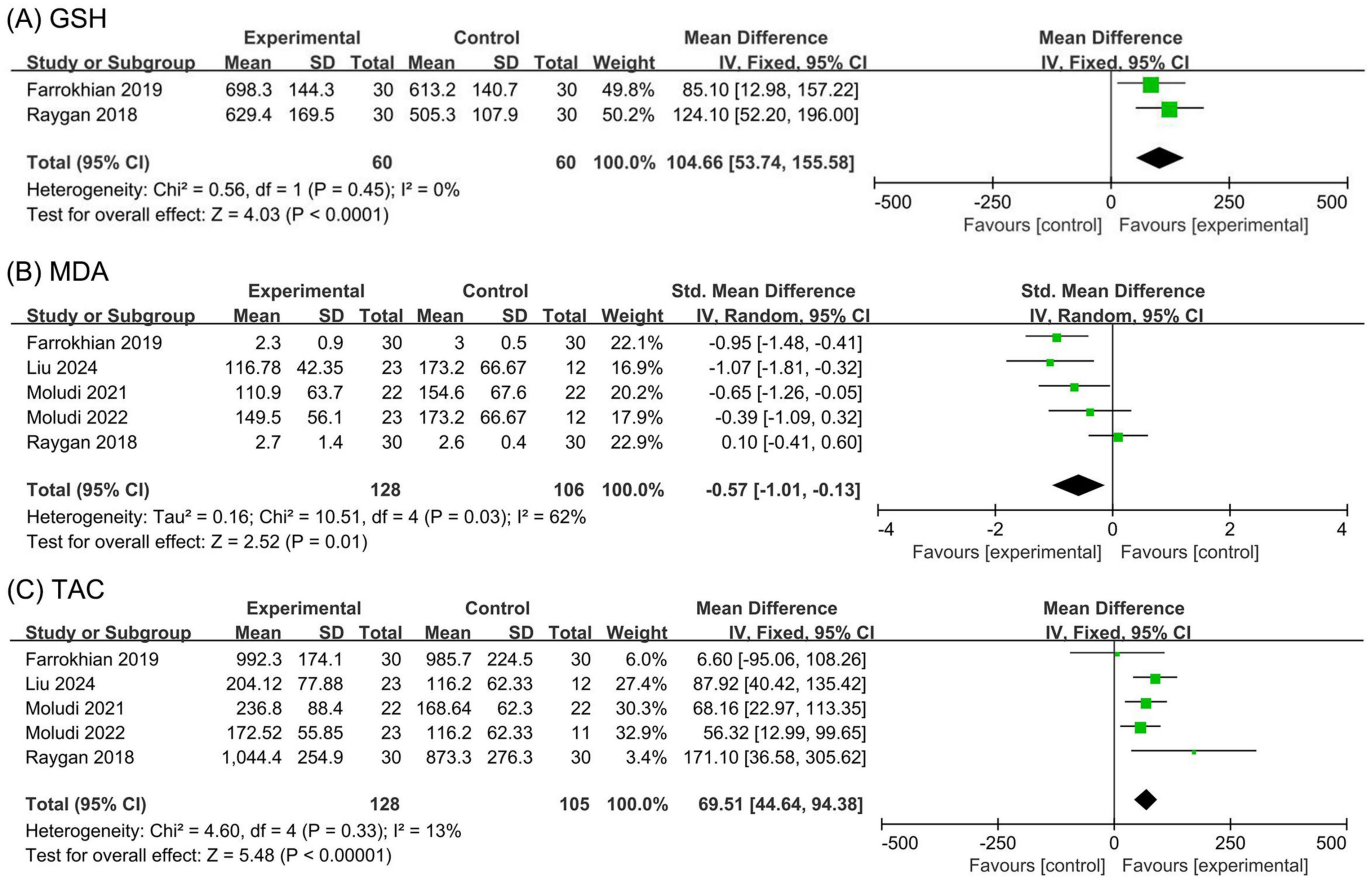
Forest plots of the meta-analysis on oxidation outcomes: **(A)** GSH; **(B)** MDA; **(C)** TAC. GSH, glutathione; MDA, malondialdehyde; TAC, total antioxidant capacity.

The meta-analysis for GSH levels included two studies (23, 28) with 120 participants. The Cochran’s *Q* test and *I*^2^ statistic indicated minor heterogeneity (*p* = 0.45, *I*^2^ = 0). The results demonstrated that the probiotic group significantly increased GSH levels compared to the placebo group (MD 104.66 μmol/L, 95% CI 53.74 to 155.58, *p* < 0.0001), as depicted in Figure 4A.

##### 3.4.2.2 MDA

The meta-analysis for MDA levels included five studies (23, 24, 26–28) with 234 participants. The Cochran’s *Q* test and *I*^2^ statistic indicated high heterogeneity (*p* = 0.03, *I*^2^ = 62%). The results suggested a moderate reduction in MDA levels in the probiotic group compared to the placebo group (SMD −0.57, 95% CI −1.01 to −0.13, *p* = 0.01), as shown in Figure 4B.

##### 3.4.2.3 TAC

The meta-analysis for TAC included five studies (23, 24, 26–28) with 233 participants. The Cochran’s *Q* test and *I*^2^ statistic indicated minor heterogeneity (*p* = 0.33, *I*^2^ = 13%). The results revealed that the probiotic group significantly increased TAC compared to the placebo group (MD 69.51 mmol/L, 95% CI 44.64 to 94.38, *p* < 0.00001), as illustrated in Figure 4C.

#### 3.4.3 Inflammatory outcomes

##### 3.4.3.1 Hs-CRP

**Figure 5:**
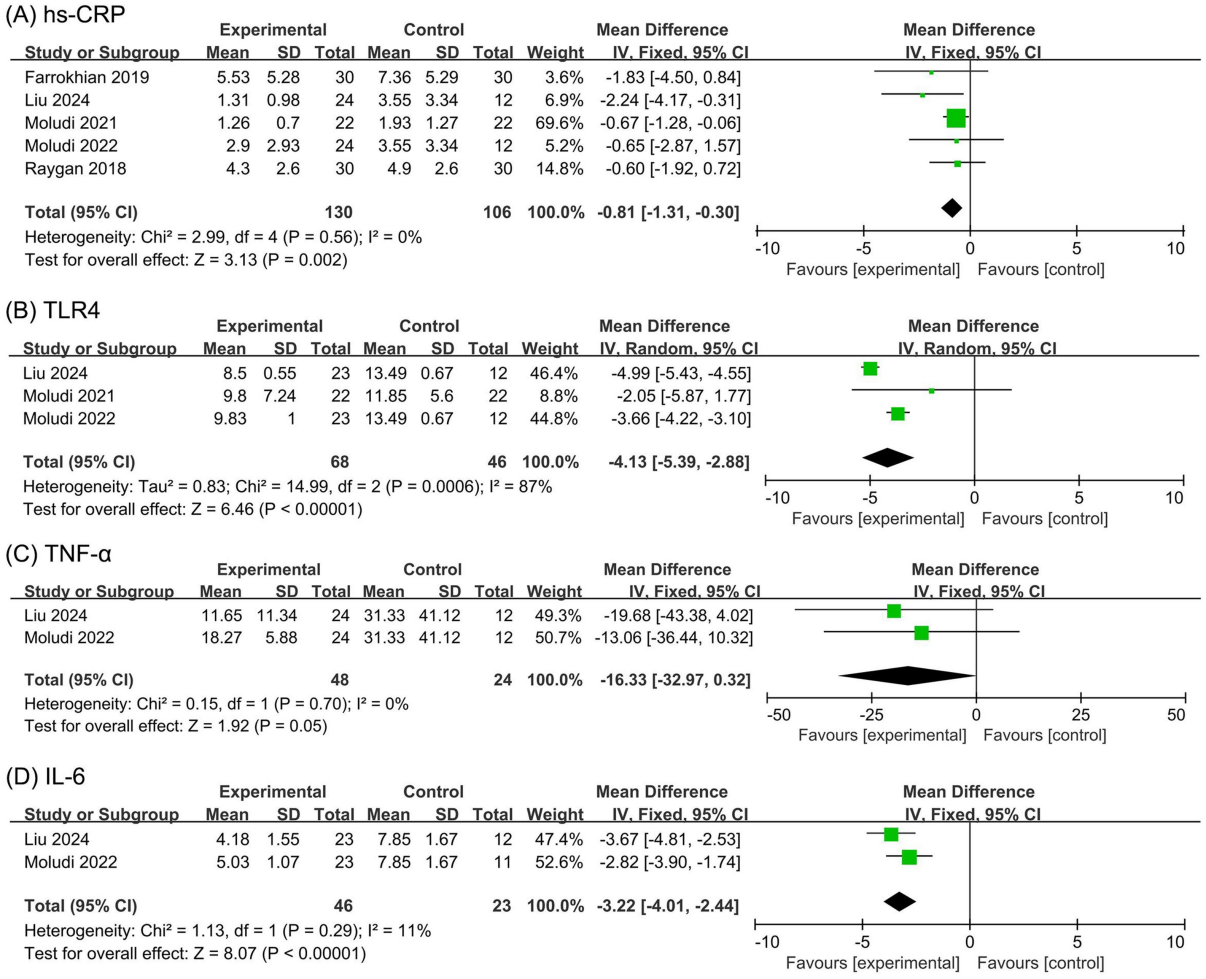
Forest plots of the meta-analysis on inflammatory outcomes: **(A)** hs-CRP; **(B)** TLR4; **(C)** TNF-α; **(D)** IL-6. hs-CRP, high-sensitivity C-reactive protein; TLR4, Toll-like receptor 4; TNF-α, tumor necrosis factor-alpha; IL-6, interleukin-6.

The meta-analysis for hs-CRP levels included five studies (23, 24, 26–28) with 236 participants. The Cochran’s *Q* test and *I*^2^ statistic indicated minor heterogeneity (*p* = 0.56, *I*^2^ = 0). The results showed a significant reduction in hs-CRP levels in the probiotic group compared to the placebo group (MD −0.81 ng/mL, 95% CI −1.31 to −0.30, *p* = 0.002), as depicted in Figure 5A.

##### 3.4.3.2 TLR4

The meta-analysis for TLR4 levels included three studies (24, 26, 27) with 114 participants. The Cochran’s *Q* test and *I*^2^ statistic indicated high heterogeneity (*p* = 0.0006, *I*^2^ = 87%). The results revealed a significant decrease in TLR4 levels in the probiotic group compared to the placebo group (MD −4.13 ng/mL, 95% CI −5.39 to −2.88, *p* < 0.00001), as shown in Figure 5B.

##### 3.4.3.3 TNF-α

The meta-analysis for TNF-α levels included two studies (24, 27) with 72 participants. The Cochran’s *Q* test and *I*^2^ statistic indicated minor heterogeneity (*p* = 0.70, *I*^2^ = 0). The results showed no significant difference in TNF-α levels between the probiotic and placebo groups (MD −16.33 ng/mL, 95% CI −32.97 to 0.32, *p* = 0.054), as illustrated in Figure 5C.

##### 3.4.3.4 IL-6

The meta-analysis for IL-6 levels included two studies (24, 27) with 69 participants. The Cochran’s *Q* test and *I*^2^ statistic indicated minor heterogeneity (*p* = 0.29, *I*^2^ = 11%). The results showed a significant decrease in IL-6 levels in the probiotic group compared to the placebo group (MD −3.22 ng/mL, 95% CI −4.01 to −2.44, *p* < 0.00001), as depicted in Figure 5D.

#### 3.4.4 Safety outcomes

##### 3.4.4.1 AER

**Figure 6:**
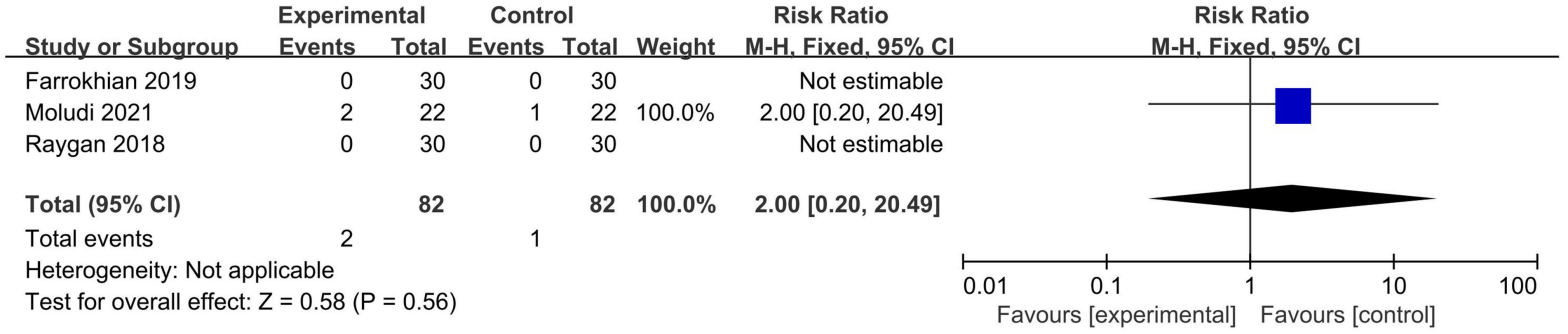
Forest plots of the meta-analysis on adverse event rate.

The meta-analysis for AER included three studies (23, 26, 28) with 164 participants. The AER in the probiotic group was 2.44%, compared to 1.22% in the placebo group. The Cochran’s *Q* test and *I*^2^ statistic indicated minor heterogeneity (*p* = 1.00, *I*^2^ = 0%). The results showed no statistically significant difference in AER between the probiotic and placebo groups (RR 2.00, 95% CI 0.20 to 20.49, *p* = 0.56), as shown in Figure 6.

### 3.5 Sensitivity analysis

The above analysis indicated that HDL-C and TC exhibited moderate heterogeneity (25% ≤ *I*^2^ < 50%), while LDL-C, MDA, and TLR4 demonstrated high heterogeneity (*I*^2^ ≥ 50%). Therefore, leave-one-out sensitivity analyses were conducted to investigate the sources of heterogeneity for these outcomes, as shown in Table 2.

**Table 2:** Leave-one-out sensitivity analyses of identified heterogeneous sources.

Outcome	Heterogeneity source	Sensitivity analysis results			Robustness
		*I*^2^/%	SMD/MD (95% CI)	*p*-value	
LDL-C	Sun et al. (29)	0	−5.90 (−13.13, 1.33)	0.11	Not robust
HDL-C	Raygan et al. (28)	0	1.67 (−0.51, 3.85)	0.13	Not robust
TC	Moludi et al. (25)	0	−1.94 (−12.41, 8.52)	0.72	Robust
TLR4	Liu et al. (24)	0	−3.63 (−4.18, −3.07)	<0.00001	Robust
MDA	Raygan et al. (28)	0	−0.78 (−1.09, 0.46)	<0.00001	Robust

SMD, standardized mean difference; MD, mean difference; CI, confidence interval; LDL-C, low-density lipoprotein cholesterol; HDL-C, high-density lipoprotein cholesterol; TC, total cholesterol; MDA, malondialdehyde; TLR4, Toll-like receptor 4.

The sensitivity analysis revealed that the heterogeneity of LDL-C originated from the study by Sun et al. (29), which may be attributed to the fact that the participants were all Chinese, whereas the participants in the other studies were Iranian. After removing this study, the heterogeneity of LDL-C decreased substantially (*I*^2^ = 0), but it no longer reached statistical significance (MD −5.90 mg/dL, 95% CI −13.13 to 1.33, *p* = 0.11), suggesting that the meta-analysis results for LDL-C were not robust.

The heterogeneity of TC was attributed to the study by Moludi et al. (25), likely due to their participants being patients with myocardial infarction (MI) after successful percutaneous coronary intervention (PCI), while other studies included stable patients with CHD. After removing this study, the heterogeneity of TC significantly decreased (*I*^2^ = 0%), and the statistical significance remained unchanged (MD −1.94 mg/dL, 95% CI −12.41 to 8.52, *p* = 0.72), suggesting that the meta-analysis results for TC were robust.

The heterogeneity of TLR4 was linked to the study by Liu et al. (24), which did not impose BMI restrictions on participants, whereas other studies required participants to have a BMI ≥25 kg/m^2^. After removing this study, the heterogeneity of TLR4 significantly decreased (*I*^2^ = 0), and the statistical significance remained unchanged (MD −3.63 ng/mL, 95% CI −4.18 to −3.07, *p* < 0.00001), suggesting that the meta-analysis results for TLR4 were robust.

The heterogeneity of MDA and HDL-C was traced back to the study by Raygan et al. (28). However, compared to other studies, the study by Raygan et al. (28) did not exhibit significant clinical or methodological heterogeneity. Therefore, this heterogeneity may be attributed to statistical heterogeneity. After excluding the study by Raygan et al. (28), the heterogeneity of MDA significantly decreased (*I*^2^ = 0), and the statistical significance remained unchanged (SMD −0.78, 95% CI −1.09 to −0.46, *p* < 0.00001), indicating that the meta-analysis results for MDA were robust. In contrast, after excluding this study, the heterogeneity of HDL-C significantly decreased (*I*^2^ = 0%), but it was no longer statistically significant (MD 1.67 mg/dL, 95% CI −0.51 to 3.85, *p* = 0.13), suggesting that the meta-analysis results for HDL-C were not robust.

Additionally, the leave-one-out sensitivity analysis demonstrated that the meta-analysis results for VLDL-C, TC, TG, GSH, MDA, TAC, hs-CRP, TLR4, TNF-α, and IL-6 were robust, while the results for LDL-C and HDL-C were not.

### 3.6 Subgroup analysis

The subgroup analysis was conducted to investigate how clinical factors such as participant source, average age, comorbidities, intervention program, probiotic dosage, and treatment duration influence the high heterogeneity observed in outcomes including LDL-C, HDL-C, TC, MDA, and TLR4 levels, as detailed in Table 3.

**Table 3:** Subgroup analyses of the high heterogeneity outcomes.

Outcome	Subject	Subgroup	Number of studies	*I*^2^/%	MD (95% CI)	*p*-value
LDL-C	Participant source	China	1	0	−18.95 (−22.75, −15.15)	<0.00001
		Iran	5	0	−5.90 (−13.13, 1.33)	0.11
	Average age	<60 years old	4	0	−8.14 (−17.30, 1.03)	0.08
		≥60 years old	2	82	−11.54 (−27.84, 4.77)	0.17
	Comorbidities	CHD	3	0	−18.50 (−22.22, −14.78)	<0.00001
		CHD combined with T2DM	2	0	−3.59 (−11.80, 4.63)	0.39
		MI after successful PCI	1	0	−26.50 (−52.77, −0.23)	0.048
	Intervention program	Synbiotics	2	0	−3.24 (−14.02, 7.54)	0.56
		Probiotics	4	53	−14.16 (−23.81, −4.51)	0.004
	Probiotic dosage	<2 × 10^9^ CFU/day	5	0	−17.37 (−20.87, −13.86)	<0.00001
		≥2 × 10^9^ CFU/day	1	82	−2.20 (−13.97, 9.57)	0.71
	Treatment duration	≤12 weeks	5	0	−5.90 (−13.13, 1.33)	0.11
		24 weeks	1	0	−18.95 (−22.75, −15.15)	<0.00001
HDL-C	Average age	<60 years old	3	0	0.50 (−2.45, 3.44)	0.74
		≥60 years old	2	6	4.27 (1.91, 6.64)	0.0004
	Comorbidities	CHD	2	0	2.01 (−2.04, 6.06)	0.33
		CHD combined with T2DM	2	6	4.27 (1.91, 6.64)	0.0004
		MI after successful PCI	1	0	−1.20 (−5.48, 3.08)	0.58
	Intervention program	Synbiotics	2	0	3.17 (0.29, 6.05)	0.03
		Probiotics	3	68	1.99 (−2.42, 6.40)	0.38
	Probiotic dosage	<2 × 10^9^ CFU/day	3	0	0.50 (−2.45, 3.44)	0.74
		≥2 × 10^9^ CFU/day	2	6	4.27 (1.91, 6.64)	0.0004
TC	Average age	<60 years old	4	39	−11.56 (−24.43, 1.31)	0.08
		≥60 years old	1	0	1.40 (−13.45, 16.25)	0.85
	Comorbidities	CHD	2	0	−36.06 (−81.98, 9.87)	0.12
		CHD combined with T2DM	2	0	−0.08 (−10.82, 10.67)	0.99
		MI after successful PCI	1	0	−31.60 (−57.89, −5.31)	0.02
	Intervention program	Synbiotics	2	0	0.02 (−14.50, 14.55)	1.00
		Probiotics	3	57	−17.87 (−43.02, 7.28)	0.16
	Probiotic dosage	<2 × 10^9^ CFU/day	3	0	−32.70 (−55.52, −9.88)	0.005
		≥2 × 10^9^ CFU/day	2	0	−0.08 (−10.82, 10.67)	0.99
MDA	Average age	<60 years old	3	0	−51.62 (−75.47, −27.76)	<0.0001
		≥60 years old	2	83	−0.32 (−1.10, 0.46)	0.42
	Comorbidities	CHD	2	40	−0.71 (−1.38, −0.05)	0.04
		CHD combined with T2DM	2	87	−0.42 (−1.45, 0.60)	0.42
		MI after successful PCI	1	0	−0.65 (−1.26, −0.05)	0.04
	Intervention program	Synbiotics	2	0	−0.99 (−1.42, −0.55)	<0.0001
		Probiotics	3	45	−0.25 (−0.59, 0.09)	0.15
	Probiotic dosage	<2 × 10^9^ CFU/day	3	0	−0.68 (−1.08, −0.29)	0.0006
		≥2 × 10^9^ CFU/day	2	87	−0.42 (−1.45, 0.60)	0.42
TLR4	Comorbidities	CHD	2	93	−4.34 (−5.64, −3.03)	<0.00001
		MI after successful PCI	1	0	−2.05 (−5.87, 1.77)	0.29
	Intervention program	Synbiotics	1	0	−4.99 (−5.43, −4.55)	<0.00001
		Probiotics	2	0	−3.63 (−4.18, −3.07)	<0.00001

MD, mean difference; CI, confidence interval; CHD, coronary heart disease; T2DM, type 2 diabetes mellitus; LDL-C, low-density lipoprotein cholesterol; HDL-C, high-density lipoprotein cholesterol; TC, total cholesterol; MDA, malondialdehyde; TLR4, Toll-like receptor 4.

#### 3.6.1 LDL-C

For participant source, a significant reduction in LDL-C was observed in Chinese participants (MD −18.95 mg/dL, 95% CI −22.75 to −15.15, *p* < 0.00001, *I*^2^ = 0), while no significant effect was found in Iranian participants (MD −5.90 mg/dL, 95% CI −13.13 to 1.33, *p* = 0.11, *I*^2^ = 0). Regarding average age, the results showed no significant effect in groups aged <60 years (MD −8.14 mg/dL, 95% CI −17.30 to 1.03, *p* = 0.08, *I*^2^ = 0) or ≥60 years (MD −11.54 mg/dL, 95% CI −27.84 to 4.77, *p* = 0.17, *I*^2^ = 82%). In terms of comorbidities, probiotics significantly reduced LDL-C in patients with CHD (MD −18.50 mg/dL, 95% CI −22.22 to −14.78, *p* < 0.00001, *I*^2^ = 0) and in those with MI after successful PCI (MD −26.50 mg/dL, 95% CI −52.77 to −0.23, *p* = 0.048, *I*^2^ = 0). However, the effect was not significant in patients with CHD combined with T2DM (MD −3.59 mg/dL, 95% CI −11.80 to 4.63, *p* = 0.39, *I*^2^ = 0). Regarding intervention program, probiotics significantly decreased LDL-C (MD −14.16 mg/dL, 95% CI −23.81 to −4.51, *p* = 0.004, *I*^2^ = 53%), whereas synbiotics showed no significant effect (MD −3.24 mg/dL, 95% CI −14.02 to 7.54, *p* = 0.56, *I*^2^ = 0). For probiotic dosage, a dose of < 2 × 10^9^ CFU/day significantly reduced LDL-C (MD −17.37 mg/dL, 95% CI −20.87 to −13.86, *p* < 0.00001, *I*^2^ = 0), whereas a dose ≥2 × 10^9^ CFU/ day had no significant effect (MD −2.20 mg/dL, 95% CI −13.97 to 9.57, *p* = 0.71, *I*^2^ = 82%). Concerning treatment duration, studies with ≤12 weeks of intervention showed no significant effect (MD −5.90 mg/dL, 95% CI −13.13 to 1.33, *p* = 0.11, *I*^2^ = 0), whereas a 24-week intervention yielded a significant reduction (MD −18.95 mg/dL, 95% CI −22.75 to −15.15, *p* < 0.00001, *I*^2^ = 0). In summary, heterogeneity in LDL-C levels appears to be associated with participant source, comorbidities, and treatment duration, rather than average age, intervention program, or probiotic dosage.

#### 3.6.2 HDL-C

For average age, a significant increase in HDL-C was observed in groups ≥60 years (MD 4.27 mg/dL, 95% CI 1.91 to 6.64, *p* = 0.0004, *I*^2^ = 0), while no significant effect was found in groups aged <60 years (MD 0.50 mg/dL, 95% CI −2.45 to 3.44, *p* = 0.74, *I*^2^ = 0). In terms of comorbidities, probiotics significantly increased HDL-C in patients with CHD combined with T2DM (MD 4.27 mg/dL, 95% CI 1.91 to 6.64, *p* = 0.0004, *I*^2^ = 6%). However, the effect was not significant in patients with CHD alone (MD 2.01 mg/dL, 95% CI −2.04 to 6.06, *p* = 0.33, *I*^2^ = 0) or MI after successful PCI (MD −1.20 mg/dL, 95% CI −5.48 to 3.08, *p* = 0.58, *I*^2^ = 0). Regarding intervention program, synbiotics significantly increased HDL-C (MD 3.17 mg/dL, 95% CI 0.29 to 6.05, *p* = 0.03, *I*^2^ = 0), whereas probiotics alone showed no significant effect (MD 1.99 mg/dL, 95% CI −2.42 to 6.40, *p* = 0.38, *I*^2^ = 68%). For probiotic dosage, a dose of ≥2 × 10^9^ CFU/day significantly increased HDL-C (MD 4.27 mg/dL, 95% CI 1.91 to 6.64, *p* = 0.0004, *I*^2^ = 6%), whereas a dose <2 × 10^9^ CFU/day had no significant effect (MD 0.50 mg/dL, 95% CI −2.45 to 3.44, *p* = 0.74, *I*^2^ = 0). In summary, heterogeneity in HDL-C levels appears to be associated with average age, comorbidities, and probiotic dosage, rather than the intervention program.

#### 3.6.3 TC

For average age, the effect was not significant in groups <60 years (MD −11.56 mg/dL, 95% CI −24.43 to 1.31, *p* = 0.08, *I*^2^ = 39%) or in groups aged ≥60 years years (MD 1.40 mg/dL, 95% CI −13.45 to 16.25, *p* = 0.85, *I*^2^ = 0). In terms of comorbidities, subgroup analyses showed that probiotics significantly reduced TC in patients with MI after successful PCI (MD −31.60 mg/dL, 95% CI −57.89 to −5.31, *p* = 0.02, *I*^2^ = 0). However, the effect was not significant in patients with CHD (MD −36.06 mg/dL, 95% CI −81.98 to 9.87, *p* = 0.12, *I*^2^ = 0) or patients with CHD combined with T2DM (MD −0.08 mg/dL, 95% CI −10.82 to 10.67, *p* = 0.99, *I*^2^ = 0). Regarding the intervention program, the effect was not significant for either synbiotics (MD 0.02 mg/dL, 95% CI −14.50 to 14.55, *p* = 1.00, *I*^2^ = 0) or probiotics (MD −17.87 mg/dL, 95% CI −43.02 to 7.28, *p* = 0.16, *I*^2^ = 57%). For probiotic dosage, a dose of <2 × 10^9^ CFU/day significantly reduced TC (MD −32.70 mg/dL, 95% CI −55.52 to −9.88, *p* = 0.005, *I*^2^ = 0), whereas a dose ≥2 × 10^9^ CFU/day had no significant effect (MD −0.08 mg/dL, 95% CI −10.82 to 10.67, *p* = 0.99, *I*^2^ = 0). In summary, heterogeneity in TC levels appears to be associated with comorbidities and probiotic dosage rather than average age or intervention program.

#### 3.6.4 MDA

For average age, a significant reduction in MDA was observed in groups <60 years years (MD −51.62 μmol/L, 95% CI −75.47 to −27.76, *p* < 0.0001, *I*^2^ = 0), whereas no significant effect was found in groups aged ≥60 years (MD −0.32 μmol/L, 95% CI −1.10 to 0.46, *p* = 0.42, *I*^2^ = 83%). In terms of comorbidities, subgroup analyses showed that probiotics significantly reduced MDA in patients with CHD (MD −0.71 μmol/L, 95% CI −1.38 to −0.05, *p* = 0.04, *I*^2^ = 40%) and in those with MI after successful PCI (MD −0.65 μmol/L, 95% CI −1.26 to −0.05, *p* = 0.04, *I*^2^ = 0). However, the effect was not significant in patients with CHD combined with T2DM (MD −0.42 μmol/L, 95% CI −1.45 to 0.60, *p* = 0.42, *I*^2^ = 87%). Regarding the intervention program, synbiotics significantly decreased MDA (MD −0.99 μmol/L, 95% CI −1.42 to −0.55, *p* < 0.0001, *I*^2^ = 0), whereas probiotics alone showed no significant effect (MD −0.25 μmol/L, 95% CI −0.59 to 0.09, *p* = 0.15, *I*^2^ = 45%). For probiotic dosage, a dose of <2 × 10^9^ CFU/day significantly decreased MDA (MD −0.68 μmol/L, 95% CI −1.08 to −0.29, *p* = 0.0006, *I*^2^ = 0), whereas a dose ≥2 × 10^9^ CFU/day had no significant effect (MD −0.42 μmol/L, 95% CI −1.45 to 0.60, *p* = 0.42, *I*^2^ = 87%). In summary, the heterogeneity of MDA levels does not appear to be explained by average age, comorbidities, the intervention program, or probiotic dosage.

#### 3.6.5 TLR4

In terms of comorbidities, subgroup analyses showed that probiotics significantly reduced TLR4 levels in patients with CHD (MD −4.34, 95% CI −5.64 to −3.03, *p* < 0.00001, *I*^2^ = 93%). However, no significant reduction was observed in patients with MI after successful PCI (MD −2.05, 95% CI −5.87 to 1.77, *p* = 0.29, *I*^2^ = 0). Regarding intervention program, both synbiotics (MD −4.99, 95% CI −5.43 to −4.55, *p* < 0.00001, *I*^2^ = 0) and probiotics (MD −3.63, 95% CI −4.18 to −3.07, *p* < 0.00001, *I*^2^ = 0) significantly decreased TLR4 levels. In summary, the heterogeneity observed in TLR4 appears to be related to differences in intervention programs rather than comorbidities.

Overall, the sensitivity and subgroup analyses indicated that the sources of heterogeneity differed across outcomes. For LDL-C, heterogeneity was mainly driven by participant source, comorbidities, and treatment duration. For HDL-C, the heterogeneity appeared to be associated with average age, comorbidities, and probiotic dosage. The heterogeneity in TC was primarily attributable to differences in comorbidities and probiotic dosage. For MDA, the main source of heterogeneity was the study by Raygan et al. (28), and subgroup analyses did not identify clear clinical factors explaining the variability. For TLR4, heterogeneity was largely linked to differences in intervention programs. These findings suggest that population characteristics, clinical conditions, intervention types, and dosage regimens collectively contribute to the heterogeneity observed across outcomes.

#### 3.6.6 TSA

**Figure 7:**
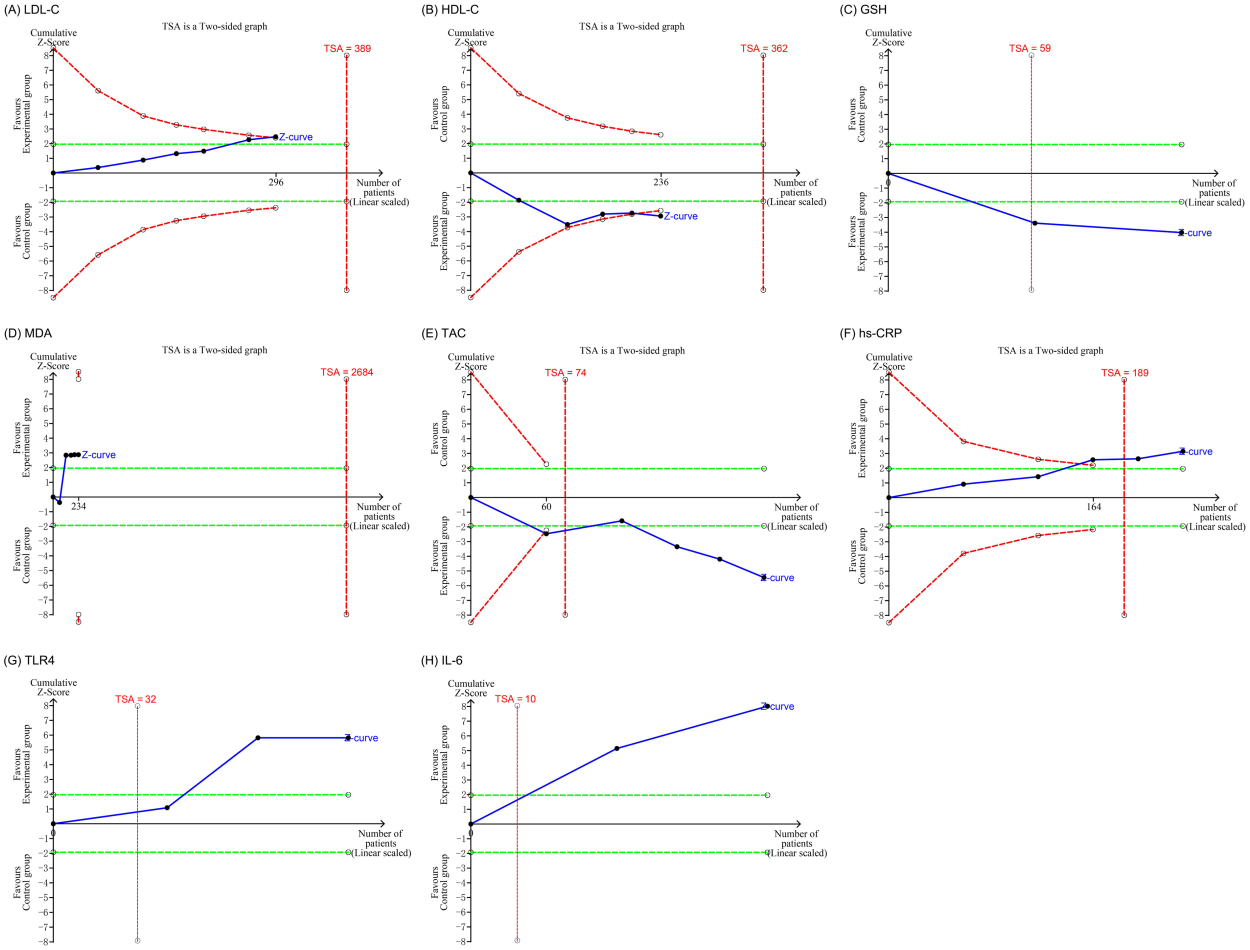
Trial sequential analyses of efficacy outcomes: **(A)** LDL-C; **(B)** HDL-C; **(C)** GSH; **(D)** MDA; **(E)** TAC; **(F)** hs-CRP; **(G)** TLR4; **(H)** IL-6. LDL-C, low-density lipoprotein cholesterol; HDL-C, high-density lipoprotein cholesterol; GSH, glutathione; MDA, malondialdehyde; TAC, total antioxidant capacity; hs-CRP, high-sensitivity C-reactive protein; TLR4, Toll-like receptor 4; IL-6, interleukin-6.

The TSA was conducted to evaluate the reliability and validity of the meta-analysis results by monitoring significant outcomes and reducing the risk of false-positive findings. The analysis showed that the *Z*-value curves for LDL-C, HDL-C, GSH, TAC, hs-CRP, TLR4, and IL-6 crossed the monitoring boundaries, suggesting that these outcomes may have reached the required information size. However, these signals should be interpreted with caution. For LDL-C and HDL-C in particular, the TSA results were inconsistent with the sensitivity analyses, which demonstrated that the pooled effects were unstable and strongly influenced by individual studies. This discrepancy indicates that crossing the TSA boundary does not guarantee clinical reliability, especially when effect sizes are small, heterogeneous, or dependent on single influential trials. In contrast, the *Z*-value curve for MDA did not cross the monitoring boundaries, indicating that this outcome requires further confirmation through additional high-quality studies (see Figure 7).

### 3.7 Publication bias

**Figure 8:**
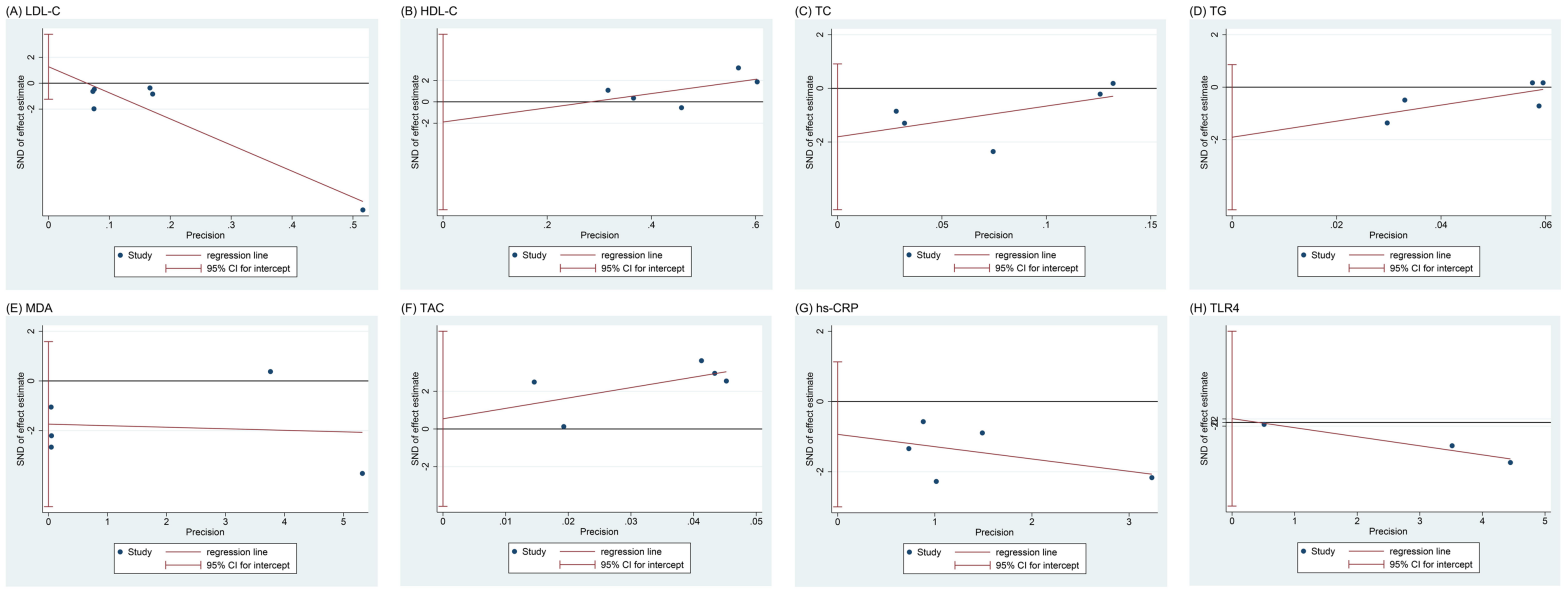
Egger’s test of publication bias: **(A)** LDL-C; **(B)** HDL-C; **(C)** TC; **(D)** TG; **(E)** MDA; **(F)** TAC; **(G)** hs-CRP; **(H)** TLR4. LDL-C, low-density lipoprotein cholesterol; HDL-C, high-density lipoprotein cholesterol; TC, total cholesterol; TG, triglycerides; MDA, malondialdehyde; TAC, total antioxidant capacity; hs-CRP, high-sensitivity C-reactive protein; TLR4, Toll-like receptor 4.

Publication bias was evaluated using Egger’s test for LDL-C, HDL-C, TC, TG, MDA, TAC, hs-CRP, and TLR4 levels, as depicted in Figure 8. The regression analysis showed no significant publication bias for these outcomes: LDL-C (*p* = 0.232), HDL-C (*p* = 0.517), TC (*p* = 0.125), TG (*p* = 0.116), MDA (*p* = 0.194), TAC (*p* = 0.735), hs-CRP (*p* = 0.245), and TLR4 levels (*p* = 0.674). Since only two studies were included for VLDL-C, GSH, TNF-α, and IL-6 levels, publication bias was not assessed for these outcomes.

### 3.8 Certainty of evidence

GRADE system indicated that the certainty of evidence for TC, TG, and TAC was moderate, reflecting a reasonable level of confidence in these estimated effects. In contrast, the certainty of evidence for LDL-C, HDL-C, VLDL-C, GSH, MDA, hs-CRP, TNF-α, IL-6, and AER was low, whereas TLR4 was very low. These ratings indicate substantial uncertainty and emphasize the necessity for further high-quality studies to validate these findings, as presented in Table 4.

**Table 4:** Certainty of evidence.

Outcome	Risk of bias	Inconsistency	Indirectness	Imprecision	Publication bias	RR/MD (95% CI)	Certainty of evidence
LDL-C	None	Serious	None	Serious	None	−11.07 (−19.94, −2.20)	Low
VLDL-C	None	None	None	Serious	Suspected	−0.93 (−5.70, 3.84)	Low
HDL-C	None	Serious	None	Serious	None	2.79 (0.95, 4.63)	Low
TC	None	None	None	Serious	None	−6.00 (−15.73, 3.72)	Moderate
TG	None	None	None	Serious	None	−6.46 (−24.15, 11.22)	Moderate
GSH	None	None	None	Serious	Suspected	104.66 (53.74, 155.58)	Low
MDA	None	Serious	None	Serious	None	−0.57 (−1.01, −0.13)	Low
TAC	None	None	None	Serious	None	69.51 (44.64, 94.38)	Moderate
hs-CRP	None	Serious	None	Serious	None	−0.81 (−1.31, −0.30)	Low
TLR4	None	Very serious	None	Serious	None	−4.13 (−5.39, −2.88)	Very low
TNF-α	None	None	None	Serious	Suspected	−16.33 (−32.97, 0.32)	Low
IL-6	None	None	None	Serious	Suspected	−3.22 (−4.01, −2.44)	Low
AER	None	None	None	Serious	Suspected	2.00 (0.20, 20.49)	Low

RR, risk ratio; MD, mean difference; CI, confidence interval; LDL-C, low-density lipoprotein cholesterol; VLDL-C, very low-density lipoprotein cholesterol; HDL-C, high-density lipoprotein cholesterol; TC, total cholesterol; TG, triglycerides; GSH, glutathione; MDA, malondialdehyde; TAC, total antioxidant capacity; hs-CRP, high-sensitivity C-reactive protein; TLR4, Toll-like receptor 4; TNF-α, tumor necrosis factor-alpha; IL-6, interleukin-6; AER, adverse event rate.

## 4 Discussion

### 4.1 Research significance and findings

To the best of our knowledge, this is the first comprehensive and methodologically rigorous meta-analysis to evaluate the effects of probiotics on lipid metabolism, inflammation, and oxidative stress in patients with CHD. Unlike previous meta-analyses, this study excluded trials combining probiotics with micronutrients, thereby ensuring greater accuracy of the results. Our analysis showed that probiotics significantly reduced MDA, hs-CRP, TLR4, and IL-6 levels, while increasing GSH and TAC levels in patients with CHD. TSA indicated that the findings were conclusive for all outcomes except MDA, further supporting the observed benefits. However, the certainty of evidence for most of these outcomes was low or very low, and these findings should therefore be interpreted with caution.

### 4.2 Efficacy of probiotics in treating CHD

As dyslipidemia is a key mechanism in the onset and progression of CHD, blood lipid levels are considered critical indicators for assessing the risk of CHD (31). This meta-analysis showed that probiotics reduced LDL-C levels and increased HDL-C levels in patients with CHD, while having no significant effects on VLDL-C, TC, or TG levels, which is consistent with the findings reported by Lei et al. (17). Conversely, an RCT by Moludi et al. (27) found that an 8-week probiotic intervention had no significant impact on TC, TG, LDL-C, or HDL-C levels in Iranian patients with CHD. We speculate that these inconsistencies may be attributed to differences in ethnicity, treatment duration, or comorbidities. Specifically, LDL-C levels showed a significant reduction in the Chinese subgroup (MD −18.95 mg/dL, 95% CI −22.75 to −15.15), whereas no significant change was observed in the Iranian subgroup (MD −5.90 mg/dL, 95% CI −13.13 to 1.33). Similarly, no significant LDL-C reduction was observed in treatment duration ≤12 weeks (MD −5.90 mg/dL, 95% CI −13.13 to 1.33), while a significant reduction was observed in the 24-week subgroup (MD −18.95 mg/dL, 95% CI −22.75 to −15.15). These findings suggest that the LDL-C-lowering effects of probiotics may require longer treatment durations or may be more pronounced in Chinese populations.

Moreover, the statistical significance of HDL-C improvements may be influenced by age, comorbidities, and probiotic formulation. Regarding age, subgroup analyses indicated that probiotics significantly increased HDL-C levels among participants aged ≥60 years, whereas no significant improvement was observed in those <60 years. In terms of comorbidities, Moludi et al. (25) reported no significant difference in HDL-C between probiotic and placebo groups among patients with CHD and obesity. In contrast, studies by Raygan et al. (28) and Tajabadi-Ebrahimi et al. (30), which focused on patients with CHD and T2DM, demonstrated significant increases in HDL-C following probiotic supplementation. These findings suggest that the HDL-C enhancing effects of probiotics may be more pronounced in patients with CHD and T2DM, which is consistent with our subgroup findings. Additionally, variations in probiotic composition and dosage may have contributed to inconsistencies across studies. Moludi et al. (25) used a single-strain formulation containing *Lactobacillus acidophilus* at a relatively low dose of 1.6 × 10^9^ CFU/day, which have limited gut colonization and metabolic effects. In contrast, Raygan et al. (28) and Tajabadi-Ebrahimi et al. (30) administered multi strain formulations containing *Bifidobacterium bifidum*, *Lactobacillus casei*, and *Lactobacillus acidophilus* at a higher dose of 2 × 10^9^ CFU/day, which may have improved colonization success and enhanced metabolic benefits. This interpretation aligns with our subgroup analysis, which showed that probiotic dose ≥2 × 10^9^ CFU/day significantly increased HDL-C levels, whereas lower doses did not.

GSH, MDA, and TAC are well-recognized biomarkers of oxidative stress, with altered levels linked to cardiovascular risk (32–34). This meta-analysis showed that probiotics supplementation significantly reduced MDA and elevated both GSH and TAC in CHD patients. Sensitivity analyses confirmed the robustness of these findings, and TSA supported the conclusiveness for GSH and TAC. These findings align with previous meta-analyses: Lei et al. (17) reported significant benefits of probiotics on GSH (SMD 0.51, 95% CI 0.03 to 0.99) and TAC (MD 104.74 mmol/L, 95% CI 42.67 to 166.81), though did not assess MDA, while Pourrajab et al. (35) observed a significant reduction in MDA in adults (MD −0.45 μmol/L, 95% CI −0.58 to −0.32). These findings suggest that probiotics can attenuate oxidative stress in CHD, consistent with experimental evidence indicating that probiotics enhance antioxidant enzyme activity, promote metabolite production, and inhibit reactive oxygen species generation (36). Notably, the effect on TAC was supported by moderate-certainty evidence, whereas evidence for GSH and MDA was of low certainty and should be interpreted cautiously.

Chronic inflammation driven by lipid metabolism is a hallmark of CHD (37), with inflammatory biomarkers such as hs-CRP, TLR4, and IL-6 are closely associated with disease activity and progression (38–41). Experimental studies have suggested probiotics modulate inflammation through immune regulation, enhanced gut barrier function, and promotion of anti-inflammatory microbial metabolites (23, 42, 43). An previous meta-analysis also support the beneficial effect of probiotics in reducing hs-CRP levels (17), consistent with our results. Notably, our study is the first to systematically demonstrate that probiotics significantly reduce TLR4, and IL-6 levels in CHD, providing new evidence for their clinical application. However, the certainty of evidence was generally low for hs-CRP, and IL-6, and very low for TLR4, suggesting that these results should be interpreted with caution. Overall, while probiotics appear to exert anti-inflammatory effects in CHD, the low to very low certainty of evidence underscores the need for well-designed, large-scale RCTs to confirm these associations and clarify strain-specific effects.

### 4.3 Safety of probiotics in treating CHD

Safety data were reported in three of the included studies. Among these, only Moludi et al. (26) noted two gastrointestinal adverse events in the probiotic group; the studies by Farrokhian et al. (23), and Raygan et al. (28) reported no adverse events. The meta-analysis revealed no significant difference in AER between probiotic and placebo groups (RR 2.00, 95% CI 0.20 to 20.49, *p* = 0.56). However, the certainty of evidence for AER was low according to the GRADE criteria, mainly due to the limited number of studies and small total sample size. Incomplete adverse events reporting and short intervention durations further limited the ability to detect rare or delayed complications. In summary, while no significant safety concerns were observed, these findings should be interpreted cautiously due to the limited evidence.

### 4.4 Selection of suitable probiotics

Based on the above analyses and discussions, inflammation and oxidative stress represent the primary pathways through which probiotics exert therapeutic effects in CHD. Therefore, probiotic formulations should prioritize strains with established anti-inflammatory and antioxidative properties. In this meta-analysis, supportive evidence derived mainly from two formulations: a single-strain preparation containing *Lactobacillus rhamnosus*, and a multi-strain preparation comprising *Bifidobacterium bifidum*, *Lactobacillus casei*, and *Lactobacillus acidophilus*. Notably, *Lactobacillus rhamnosus* has been reported to exert broad anti-inflammatory and antioxidative effects in CHD patients, significantly improving inflammatory biomarkers such as hs-CRP, TLR4, and IL-6, as well as oxidative stress markers including GSH, MDA, and TAC (24, 27). Experimental studies have shown that this strain attenuates intestinal inflammation and enhances gut barrier repair by inducing STING-dependent IL-10 production in monocytes, thereby reducing translocation of harmful gut-derived metabolites linked to endothelial and myocardial injury (44). Moreover, *Lactobacillus rhamnosus* activates the nuclear factor erythroid 2–related factor 2 signaling pathway, mitigating myocardial oxidative damage (45). These findings collectively support *Lactobacillus rhamnosus’* potential as a preferred probiotic strain in CHD management.

### 4.5 Discoveries and implications

This meta-analysis provides several novel insights into the role of probiotics in CHD management. First, unlike the meta-analysis by Lei et al. (17), our findings do not support a consistent benefit of probiotics on LDL-C and HDL-C levels across all populations. Instead, we clarified that the lipid-modulating effects of probiotics are minimal, as the observed changes did not reach the MCID. These effects appear to be influenced by specific factors such as ethnicity, treatment duration, comorbid conditions, and probiotic formulation. This finding offers a new perspective, suggesting that the cardioprotective effects of probiotics may not primarily rely on the regulation of lipid metabolism. Second, this is the first meta-analysis to evaluate the effects of probiotics on MDA levels in patients with CHD, revealing a moderate reduction. This finding provides new support for the antioxidative potential of probiotics. Third, we are also the first to systematically assess the effects of probiotics on key inflammatory markers, including TLR4 and IL-6, demonstrating that probiotics exert significant anti-inflammatory effects in patients with CHD. Together, these findings indicate that the primary mechanisms underlying the beneficial effects of probiotics on CHD prognosis may involve the regulation of inflammation and oxidative stress rather than lipid metabolism. Additionally, the safety analysis showed that probiotic supplementation did not increase the risk of adverse events in CHD patients, although this finding should be further validated in large-scale, multicenter randomized controlled trials. In summary, probiotics may represent a promising adjunctive therapy for CHD, particularly for improving inflammatory and oxidative profiles. Their clinical value may be greatest in specific patient subgroups, such as those receiving longer treatment durations or those with comorbid diabetes.

### 4.6 Limitations and prospects

This meta-analysis has several important limitations. First, none of the included trials adequately monitored or controlled participants’ dietary intake or background medications, particularly statin use. Variations in diet, especially fiber and prebiotic intake, may alter the availability of microbial substrates and thereby modify the effectiveness of probiotic supplementation. Likewise, widespread use of statins among CHD patients may mask or confound the modest lipid and inflammation effects of probiotics. The lack of standardized in reporting on these variables constitutes substantial clinical heterogeneity and limits the interpretability of the results. Second, the number of eligible studies was small, with only eight RCTs involving 296 participants. The limited sample size reduced statistical power and evidence certainty. Third, seven of the eight trials were conducted in Iran, limiting the generalizability of the results to other populations. Fourth, most interventions lasted 12 weeks or less, which may be insufficient to assess meaningful lipid changes or long-term cardiovascular outcomes. Fifth, sensitivity analyses showed that lipid outcomes (LDL-C and HDL-C) were unstable and influenced by individual studies, highlighting uncertainty of probiotics’ lipid-modulating effects. Finally, although probiotics showed anti-inflammatory and antioxidant benefits, the optimal strains, dosages, and treatment durations for CHD remain unclear.

Future research should address these limitations through large-scale, multicenter RCTs with rigorous monitoring or standardization of dietary intake and background medications. Longer intervention periods and comprehensive reporting of strain-specific responses, dosages, and co-administered therapies are essential to establish evidence-based probiotic strategies for CHD management.

## 5 Conclusion

Probiotics may offer moderate anti-inflammatory and antioxidative benefits for patients with CHD, supporting their potential as a complementary therapeutic option. However, their influence on lipid metabolism remains uncertain and clinically negligible, as the observed changes in LDL-C and HDL-C were unstable and did not reach MCID. Moreover, the low evidence certainty, geographic limitations of included trials, small sample sizes, short intervention durations, variability in probiotic formulations, and limited assessment of diet and background medications all restrict the reliability of these findings. Future large-scale, multicenter, long-term, and rigorously designed RCTs are needed to better evaluate the therapeutic potential and clinical applicability of probiotics in CHD, with particular attention to dietary context, background medications, and strain-specific effects.

## Data Availability

The original contributions presented in the study are included in the article/supplementary material, further inquiries can be directed to the corresponding authors.

## Glossary

CHD: Coronary heart disease
TSA: Trial sequential analysis
MD: Mean difference
SMD: Standardized mean difference
RR: Risk ratio
LDL-C: Low-density lipoprotein cholesterol
TAC: Total antioxidant capacity
HDL-C: High-density lipoprotein cholesterol
hs-CRP: High-sensitivity C-reactive protein
T2DM: Type 2 diabetes mellitus
VLDL-C: Very low-density lipoprotein cholesterol
TC: Total cholesterol
TG: Triglycerides
GSH: Glutathione
MDA: Malondialdehyde
TLR4: Toll-like receptor 4
TNF-α: Tumor necrosis factor-alpha
IL-6: Interleukin-6
AER: Adverse event rate
RCTs: Randomized controlled trials
CI: Confidence interval
GRADE: Grading of Recommendations, Assessment, Development, and Evaluation
MCID: Minimal clinically important difference
MI: Myocardial infarction
PCI: Percutaneous coronary intervention
NLRP3: NOD-like receptor family pyrin domain-containing 3
