# Supplementary figures and images for: Effects of probiotics on lipid metabolism, oxidation, and inflammation in coronary heart disease: a systematic review and meta-analysis
Source: Front Nutr. 2026 Jan 16;12:1681230. doi: 10.3389/fnut.2025.1681230 (PMC12858250; doi:10.3389/fnut.2025.1681230)

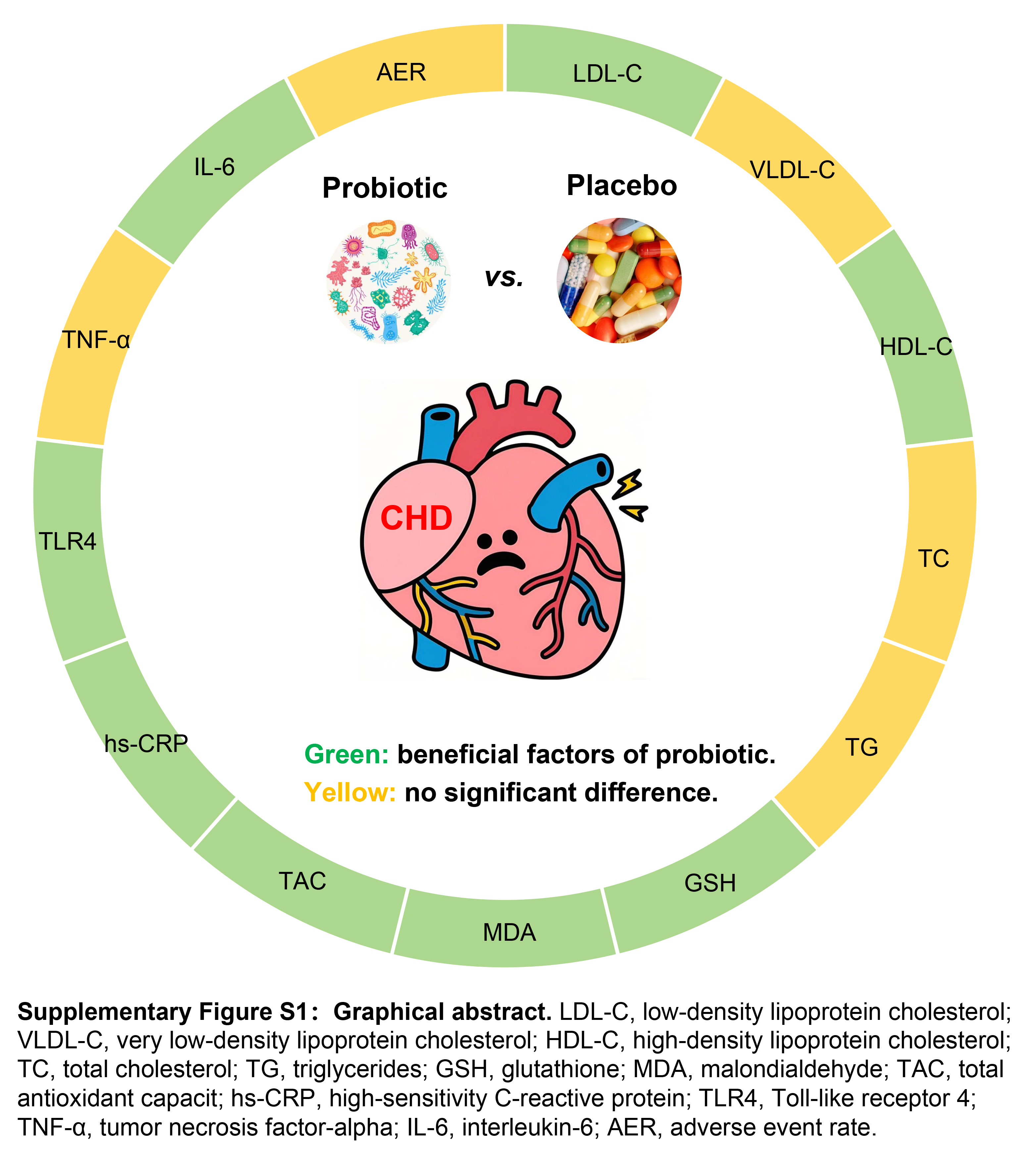

Supplement: Supplementary file 1 [file Supplementary_file_1.jpg]
